# Randomized trial of thulium laser-assisted tumor bed coagulation versus conventional suture renorrhaphy in laparoscopic partial nephrectomy: impact on perioperative outcomes and fibrosis biomarker response

**DOI:** 10.1007/s00345-025-06074-6

**Published:** 2025-12-17

**Authors:** Ehab Atallah, Abdullah Dawoud, Abul-fotouh Ahmed, Abdelrahman Ebeid, Ahmed Soliman, Hassan Abdelazim, Awatef Soliman, Samar Abdelhamid, Aly M. Abdel-karim

**Affiliations:** 1 Department of Urology, Faculty of Medicine, Al-Azhar University, Cairo, 11633 Egypt; 2 Department of Clinical Pathology, Abukhalifa Emergency Hospital, Egyptian Healthcare Insurance Authority, Ismailia, Egypt; 3 Department of Clinical Pathology, Faculty of Medicine for Girls, Al-Azhar University, Cairo, Egypt; 4 Department of Urology, Faculty of Medicine, Alexandria University, Alexandria, Egypt

**Keywords:** Laparoscopic partial nephrectomy, Thulium laser, Renorrhaphy, Warm ischemia, Renal function, Urinary biomarkers

## Abstract

**Purpose:**

This study aimed to determine whether thulium laser–assisted tumor bed hemostasis could provide effective bleeding control comparable to conventional suture renorrhaphy while minimizing ischemic exposure, parenchymal trauma, and profibrotic response. Specifically, we compared perioperative outcomes, renal functional preservation, and urinary fibrosis biomarker profiles between the two techniques during laparoscopic partial nephrectomy (LPN).

**Methods:**

In this prospective randomized trial (March 2024–September 2025), 48 patients with clinical T1 renal masses and a normal contralateral kidney were assigned to thulium laser-assisted LPN (LLPN) or conventional LPN (CLPN). In LLPN, tumor bed hemostasis was achieved with a 2-μm continuous thulium laser followed by cortical closure, while CLPN employed standard medullary and cortical renorrhaphy. Perioperative outcomes, renal function (estimated glomerular filtration rate, eGFR), and urinary fibrosis biomarkers (transforming growth factor-β1 [TGF-β1], monocyte chemoattractant protein-1 [MCP-1]) were assessed preoperatively and on postoperative day 1, and at 1 and 3 months.

**Results:**

Forty-three patients (LLPN 23, CLPN 20) were analyzed. Operative time and warm ischemia time were shorter in LLPN than CLPN (95 vs. 121 min, *p* = 0.001; 13 vs. 17 min, *p* = 0.037). LLPN required fewer sutures (8 vs. 14, *p* < 0.001) and resulted in lower blood loss (150 vs. 300 mL, *p* < 0.001). All complications were low-grade and comparable. eGFR declined significantly in both groups, with partial recovery in LLPN but not CLPN; between-group differences were not significant. Urinary TGF-β1 and MCP-1 increased postoperatively in both groups but were consistently lower in LLPN at all time points (*p* < 0.05).

**Conclusion:**

Thulium laser coagulation during LPN reduces ischemia, blood loss, and suture requirement without compromising safety or short-term renal function. The attenuated rise in fibrosis biomarkers suggests a potential long-term nephron-sparing advantage.

The study was registered on ClinicalTrials.gov (Identifier: NCT06322745, registered March 14, 2024).

## Introduction

The incidence of small renal masses (SRMs) has increased with the widespread use of advanced imaging [1]. Partial nephrectomy (PN) is the gold-standard treatment, achieving oncologic outcomes comparable to radical nephrectomy while better preserving renal function [2–4]. Minimally invasive approaches, including robotic and laparoscopic PN (LPN), further improve perioperative outcomes by reducing blood loss, hospital stay, and recovery time [5]. A persistent challenge, however, is the need for renal ischemia to ensure hemostasis [6].

Conventional hemostasis during LPN relies on double-layer suture renorrhaphy. While effective, this technique prolongs ischemia, sacrifices parenchyma, and requires advanced laparoscopic skill [7]. Beyond securing hemostasis and closing the collecting system, sutures may compromise vascularized tissue and contribute to long-term functional decline [8].

Laser-based coagulation has emerged as an alternative to minimize parenchymal loss. The 2-μm continuous thulium laser provides precise cutting and coagulation with minimal collateral damage due to its shallow penetration (~ 0.2 mm) [9–11]. It has been successfully applied in benign prostatic hyperplasia, bladder tumors, and lithotripsy, with emerging evidence supporting its use in renal surgery. Early reports suggest that thulium laser-assisted LPN (LLPN), even in off-clamp settings, is safe and feasible for small exophytic renal tumors [12].

Healing after PN involves both acute and chronic injury at the tumor bed. Renorrhaphy may exacerbate chronic ischemia and fibrotic remodeling through extracellular matrix deposition [13]. Biomarkers such as transforming growth factor-β (TGF-β) and monocyte chemoattractant protein-1 (MCP-1) are recognized indicators of renal fibrosis and predictors of functional decline [14, 15].

We hypothesized that thulium laser coagulation achieves hemostasis comparable to suture renorrhaphy while reducing ischemia, parenchymal injury, and fibrogenic activity. The objective of this study was to compare perioperative, functional, and biomarker outcomes between the two techniques. To our knowledge, this is the first randomized trial directly comparing thulium laser coagulation and conventional suture renorrhaphy for tumor bed hemostasis in LPN.

## Patients and methods

### Study population

This study was conducted at two tertiary care centers between March 2024 and September 2025. Consecutive adults with clinical T1 renal masses and a normal contralateral kidney were eligible. Exclusion criteria were hilar tumors, American Society of Anesthesiologists (ASA) score ≥ 3, RENAL nephrometry score > 9, or pre-existing chronic kidney disease (CKD).

All patients underwent baseline clinical assessment, laboratory testing, and imaging. Renal function was evaluated using serum creatinine and estimated glomerular filtration rate (eGFR), calculated with the Modification of Diet in Renal Disease (MDRD) equation, and CKD stage was classified according to Kidney Disease: Improving Global Outcomes (KDIGO) criteria. Preoperative urinary fibrosis biomarkers (TGF-β1 and MCP-1), were assessed in morning urine samples collected on the day of surgery to establish baseline levels. Tumor characterization and staging were performed with contrast-enhanced abdominal computed tomography (CT).

After written informed consent, patients were randomized (1:1) to either thulium laser–assisted LPN (LLPN) or conventional LPN (CLPN). Randomization was stratified by RENAL score (≤ 6 vs > 6) and tumor size (≤ 40 mm vs > 40 mm), with each center using its own allocation list. Patients, the allocator, and investigators responsible for biomarker analysis were blinded to group assignments throughout the study.

The sample size was estimated using G*Power software. Assumptions included a 4-min reduction in warm ischemia time (WIT; SD 4.0 within each group) and a 10% increase in biomarker change from baseline (SD 10 within each group) with LLPN. Based on these parameters, 38 patients (19 per group) were required to achieve 85% power at a two-sided α of 0.05. To compensate for potential dropouts, the planned enrollment was increased to 48 patients (24 per group). The study was approved by the Institutional Research Ethics Committee (Approval No.: URO-Azh 2023-18) and registered at ClinicalTrials.gov (NCT06322745).

### Surgical procedures

All procedures were performed under general anesthesia via a transperitoneal approach with the patient placed in the modified lateral kidney position. The renal hilum was carefully dissected using advanced vessel-sealing devices (LigaSure® or EnSeal®), and the renal artery was selectively clamped with laparoscopic bulldog clamps to achieve warm ischemia. Tumor margins were delineated, and tumor enucleation was performed using cold scissors with a combination of sharp and blunt dissection, maintaining a minimal parenchymal margin of approximately 2 mm.

**Fig. 1:**
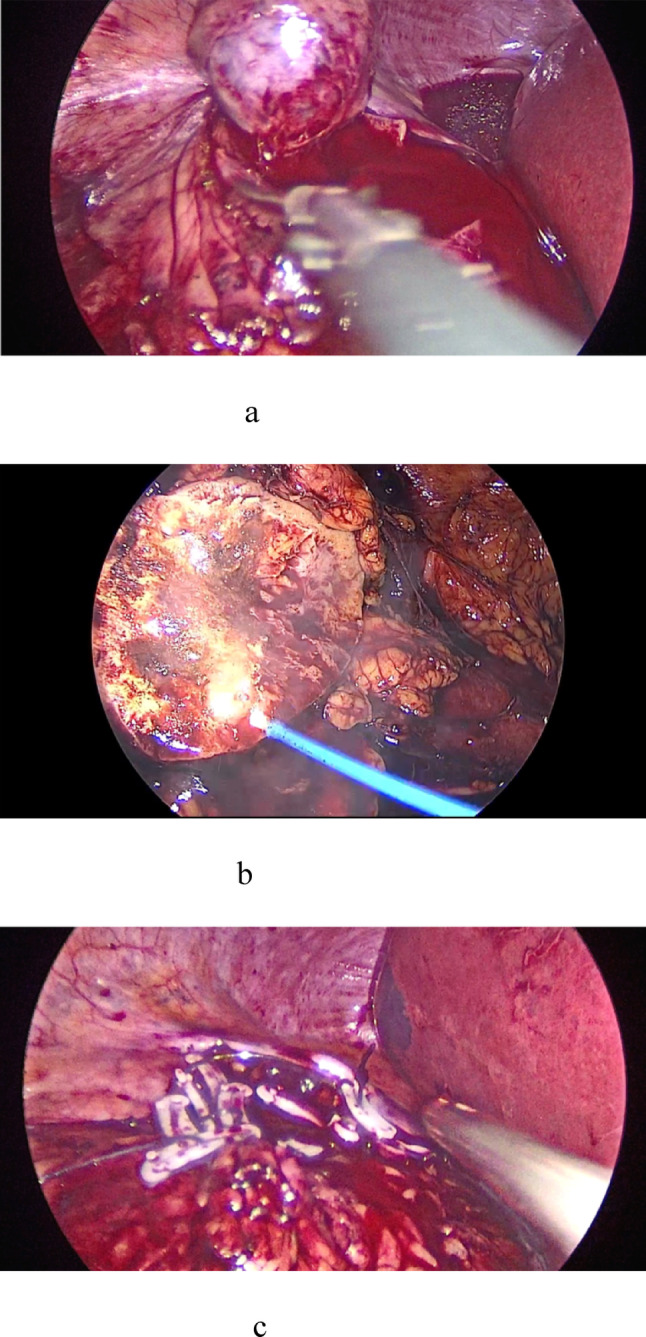
Laparoscopic partial nephrectomy with thulium laser–assisted tumor bed hemostasis. **a** Tumor enucleation with preservation of peritumor tissue. **b** Medullary bed coagulation using a 2-μm continuous thulium laser (RevoLix® 200 W, 800-μm fiber, 80 W). **c** Cortical renorrhaphy following laser coagulation, performed with V-Loc™ barbed sutures and hem-o-lok clips (sliding clip technique)

In LLPN, medullary hemostasis was achieved using a 2-µm continuous thulium laser (RevoLix® 200 W; LISA Laser Products, Germany) delivered through an 800-µm fiber at 80 W. Cortical closure was then performed with 2–0 or 3–0 V-Loc™ barbed sutures, secured with hem-o-lok clips (sliding-clip technique) (Fig. 1). In CLPN, both medullary and cortical layers were closed with the same materials and technique.

In both groups, adjunctive hemostatic agents were used as needed, and the renal artery was unclamped early once medullary hemostasis was achieved. All procedures were performed by the same surgical team (AA, EA, AD, AS).

During hospitalization, patients were closely monitored. A complete blood count (CBC) was obtained within the first 24 h, and on postoperative day 1 (POD1), renal function tests (serum creatinine and eGFR), urinary fibrosis biomarkers (TGF-β1 and MCP-1), and renal ultrasound were performed. After discharge, outpatient follow-up visits were scheduled at 1 month (POM1) and 3 months (POM3) to reassess renal function and urinary biomarkers. At POM3, a contrast-enhanced abdominal computed tomography scan was performed to evaluate for residual tumor or recurrence.

### Study endpoints

The primary endpoints were WIT and fibrogenic response, assessed by changes in urinary TGF-β1 and MCP-1 levels. Secondary endpoints included intraoperative blood loss, operative time, renorrhaphy time, number of sutures, time to ambulation, duration of hospital stay, and short-term renal functional preservation, evaluated by changes in eGFR and CKD stage during follow-up. Surgical complications were recorded and graded according to the Clavien–Dindo classification [16].

### Statistical analysis

All analyses were conducted using IBM SPSS Statistics, version 25 (IBM Corp., Armonk, NY, USA). Categorical variables were summarized as numbers and percentages and compared with Fisher’s exact test. Continuous variables were tested for normality with the Shapiro–Wilk test and reported as medians with the interquartile ranges (IQR: 25th, 75th percentiles). Between-group comparisons utilized the Mann–Whitney U test. Changes within the group from baseline to follow-up were assessed using the Wilcoxon signed-rank test, and data at multiple time points were analyzed with the Friedman test. Correlations between continuous variables were evaluated using Spearman’s rank correlation. All tests were two-tailed, with statistical significance set at p < 0.05.

## Results

**Fig. 2:**
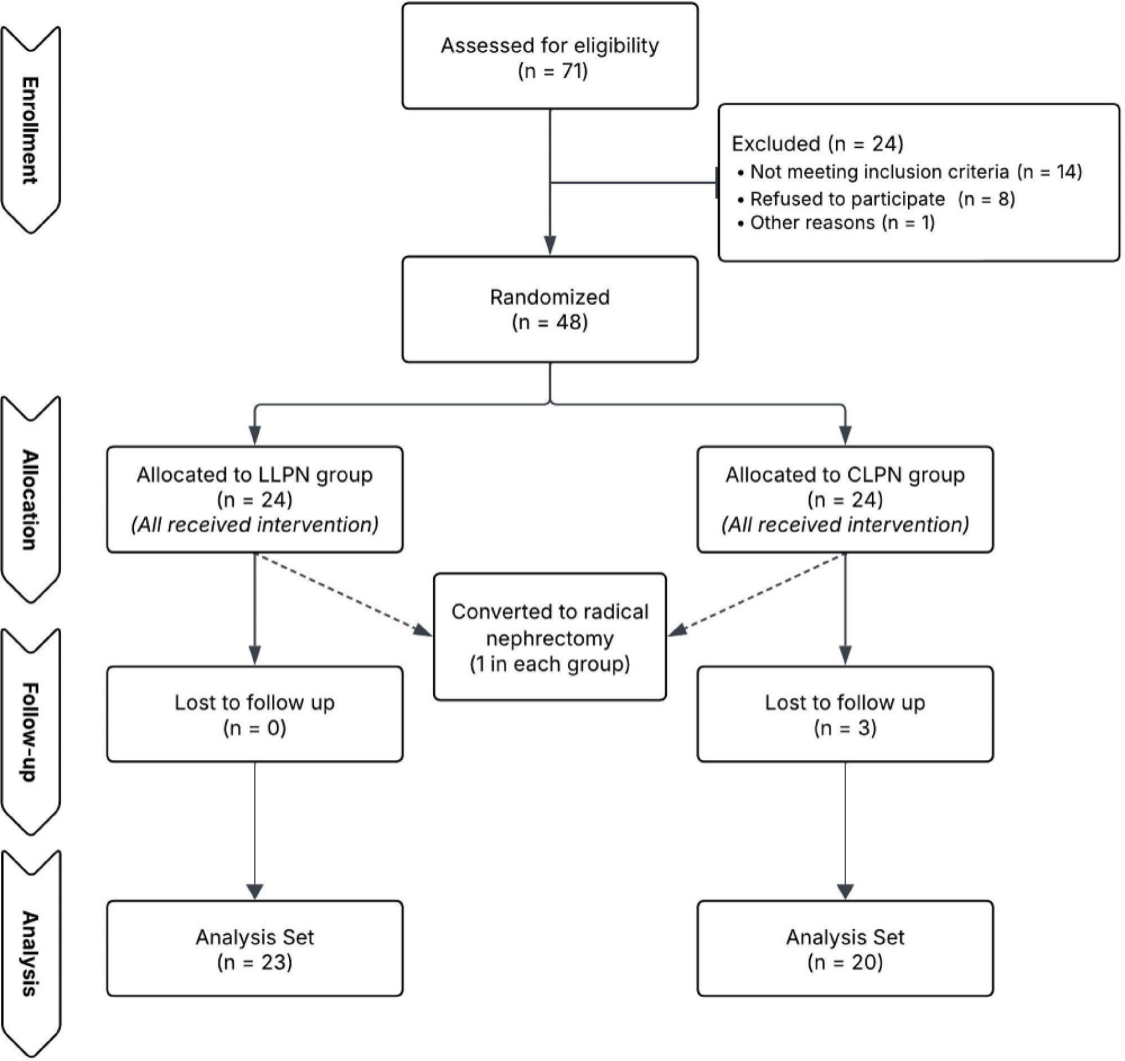
CONSORT flow diagram of patient enrollment, randomization, and analysis

A total of 48 patients were enrolled, 24 randomized to each group. One patient in each group was converted to radical nephrectomy, and three in the CLPN group had incomplete postoperative data. Thus, 23 patients in the LLPN group and 20 in the CLPN group were included in the final analysis (Fig. 2).

The median (IQR) age was 47 (38–56) years, clinical tumor size 42 (35–53) mm, and tumor complexity, as assessed by RENAL score, 8 (5–9). Overall, 62.8% of the patients were male, with 21 (48.8%) having T1a tumors and 22 (51.2%) having T1b tumors. The baseline demographic, clinical, laboratory, and tumor characteristics were comparable between the groups, except for tumor laterality (P = 0.015) (Table 1).

**Table 1:** Baseline patient and tumor characteristics

Variables	LLPN group (n = 23)	CLPN group (n = 20)	p-value
Patients clinical features			
Age, years	52 (40, 57)	45 (23, 52)	0.167
Male gender	12 (52.2)	15 (75.0)	0.122
BMI, kg/m^2^	33 (28, 44)	31 (30, 33)	0.173
Diabetes mellitus	5 (21.7)	3 (15.0)	0.704
Hypertension	9 (39.1)	5 (25.0)	0.353
CCI	3 (2, 5)	2 (2, 3)	0.104
Hemoglobin, g/dL	13 (13, 14)	14 (13, 15)	0.300
Serum creatinine, µmol/L	97 (79, 114)	93 (69, 102)	0.396
Tumor features			
Side			0.015
RT kidney	10 (43.5)	16 (80.0)	
LT kidney	13 (56.5)	4 (20.0)	
Largest diameter, mm	42 (35, 51)	41 (25, 54)	0.716
Growth pattern			0.692
Mainly exophytic (> 50%)	9 (39.1)	10 (50.0)	
Mainly endophytic (> 50%)	10 (43.5)	8 (40.0)	
Completely endophytic	4 (17.4)	2 (10.0)	
cTNM stage			0.887
1a	11 (47.8)	10 (50.0)	
1b	12 (52.2)	10 (50.0)	
RENAL score	6.5 (2.1)	7.7 (1.9)	0.051

Continuous data are presented as medians (interquartile ranges) and categorical data as numbers (percentages)

*BMI* body mass index, *CCI* Charlson Comorbidity Index, *cTNM* clinical tumor–node–metastasis

### Perioperative outcomes

Perioperative outcomes are summarized in Table 2. Median (IQR) overall operative time and WIT were significantly shorter with the LLPN group compared to the CLPN group (95 [90–100] vs 121 [105–150] minutes, P = 0.001) and (13 [10–15] vs 17 [13–22] minutes, P = 0.037). The LLPN group required fewer sutures (8 [8–10] vs 14 [12–16], P < 0.001) and experienced lower EBL (150 [100–195] vs 300 [150–550] mL, P < 0.001). All complications were low-grade. Grade II events included paralytic ileus in two CLPN patients and one LLPN patient, and one blood transfusion in the CLPN group. The complication rates, time to ambulation, and length of hospital stay were similar between the groups. All cases were confirmed as clear cell renal carcinoma, with no positive surgical margins or radiologic evidence of recurrence reported during follow-up.

**Table 2:** Perioperative outcomes

Variables	LLPN group (n = 23)	CLPN group (n = 20)	p-value
Operative time, minutes	95 (90, 100)	121 (105, 150)	0.001
Ischemia time, minutes	13 (10, 15)	17 (13, 22)	0.011
Renorrhaphy time, minutes	9 (7, 11)	15 (13, 17)	< 0.001
Number of sutures	8 (8, 10)	14 (12, 16)	< 0.001
Coagulation time, minutes	5 (5, 7)	-	-
EBL, mL	150 (100, 195)	300 (150, 550)	0.011
Hemoglobin reduction, g/dL	1 (0.6, 1.2)	1.4 (0.6, 2.7)	0.068
Intraoperative complications	0 (0.0)	0 (0.0)	-
Postoperative complications			
Grade 1	11 (100)	9 (80.0)	> 0.999
Grade 2	1 (0.0)	3 (20.0)	0.323
Paralytic ileus	1 (4.3)	2 (10.0)	
Blood transfusion	0 (0.0)	1 (5.0)	
Time to mobilization, days	1 (1, 1)	1 (1, 1)	0.855
Hospital stays, days	2 (2, 2)	2 (2, 3)	0.073

Continuous data are presented as medians (interquartile ranges) and categorical data as numbers (percentages)

*EBS* estimated blood loss

### Renal functional outcomes and urinary biomarker responses

**Fig. 3:**
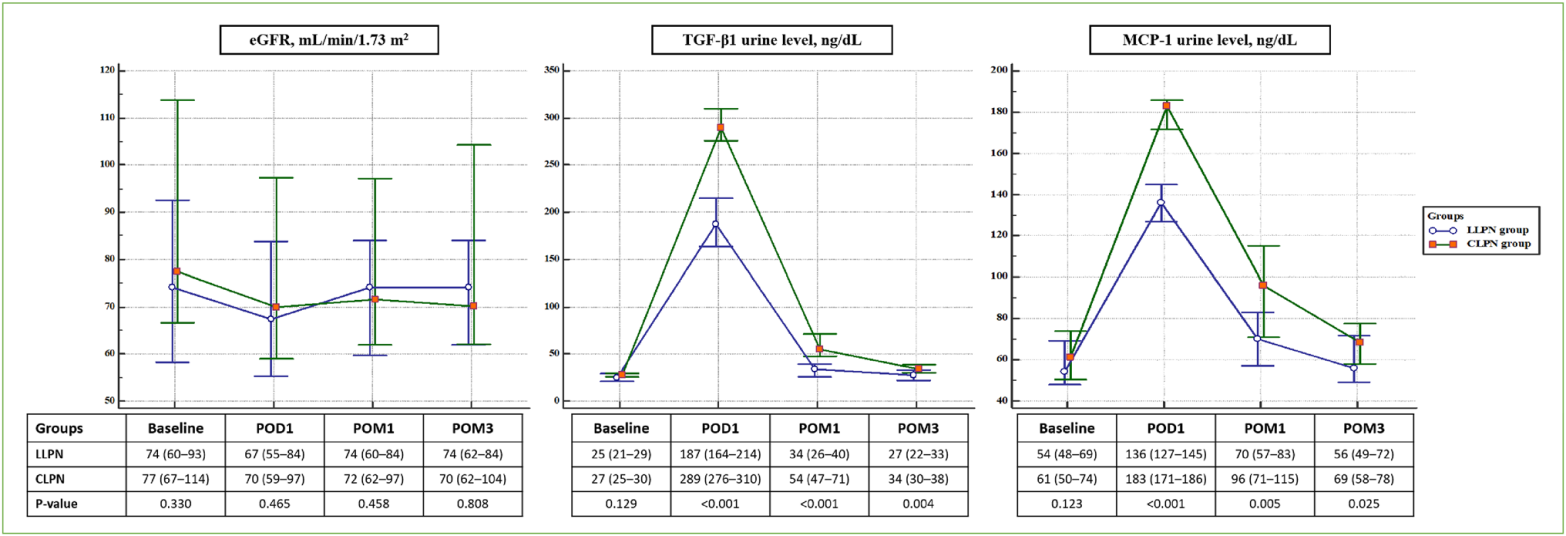
Percent changes from baseline in estimated glomerular filtration rate and urinary fibrosis biomarker levels, shown as medians (interquartile ranges) across follow-up time points . *eGFR* estimated glomerular filtration rate, *MCP-1* monocyte chemoattractant protein 1, *POD* postoperative day, *POM* postoperative month, *TGF-β1* transforming growth factor beta 1

eGFR decreased significantly on POD1 in both groups and remained below baseline at POM3 (LLPN: p = 0.003; CLPN: p < 0.001). Partial recovery was observed from POM1 in the LLPN group (p = 0.032), whereas no significant improvement occurred in the CLPN group (p = 0.078). Between-group differences were not significant at any time point. Urinary TGF-β1 and MCP-1 levels rose significantly in both groups, peaking on POD1 (all p < 0.001 vs baseline). Although values declined at POM1 and POM3, they remained significantly elevated compared with baseline (p < 0.001). Median levels were significantly higher in the CLPN group at all follow-up time points (Fig. 3).

Percent changes from baseline were analyzed for both renal function and fibrosis biomarkers. Relative eGFR reduction and KDIGO upstaging were comparable between groups at all follow-up points, and no patient developed AKI, defined as a ≥ 25% postoperative decline in eGFR. By contrast, percent increases in fibrosis biomarkers were consistently greater in the CLPN group throughout follow-up (Table 3).

**Table 3:** Renal functional outcomes and urinary biomarker responses

Variables	LLPN group (n = 23)	CLPN group (n = 20)	p-value
%Δ eGFR, mL/min/1.73 m^2^			
POD1	-9 (-10, -8)	-15 (-17, -5)	0.295
POM1	-6 (-9, -2)	-8 (-13, 0)	0.232
POM3	-4 (-9, 0)	-5 (-14, -2)	0.168
KDIGO upstaging			
POD1	9 (39.1)	8 (40.0)	> 0.999
POM1	6 (26.1)	8 (40.0)	0.515
POM3	4 (17.4)	6 (30.0)	0.473
%Δ TGF-β1 urine level, ng/dL			
POD1	+ 678 (+ 580, + 733)	+ 974 (+ 841, + 1110)	< 0.001
POM1	+ 24 (+ 15, + 40)	+ 102 (+ 66, + 170)	< 0.001
POM3	+ 6 (+ 3, + 10)	+ 19 (+ 9, + 25)	0.002
%Δ MCP-1 urine level, ng/dL			
POD1	+ 128 (+ 95, + 194)	+ 194 (+ 140, + 225)	0.009
POM1	+ 31 (+ 14, + 37)	+ 48 (+ 25, + 74)	0.004
POM3	+ 3 (0, + 5)	+ 7 (+ 3, + 15)	0.005

Continuous data are presented as medians (interquartile ranges) and categorical data as numbers (percentages)

*%Δ* percent difference from baseline, *eGFR* estimated glomerular filtration rate, *KDIGO* Kidney Disease: Improving Global Outcomes, *MCP-1* monocyte chemoattractant protein 1, *POD* postoperative day, *POM* postoperative month, *TGF-β1* transforming growth factor beta 1

Across the entire cohort (n = 43), Spearman’s correlation analysis showed no significant association between WIT and the percent change in fibrosis biomarkers at 3 months (TGF-β1: r = 0.185, p = 0.235, and MCP-1: r = –0.148, p = 0.344).

Perioperative, renal functional, and biomarker outcomes by tumor laterality are summarized in Table 4. Statistical comparisons were limited to left-sided tumors, as the right-sided subgroup was underpowered for inferential testing.

**Table 4:** Perioperative, renal functional, and biomarker outcomes by tumor laterality in the LLPN and CLPN groups

	Right-sided tumors		Left-sided tumors		
	LLPN group (n = 4)	CLPN group (n = 13)	LLPN group (n = 10)	CLPN group (n = 16)	p-value
Operative time, minutes	90 (90, 100)	120 (100, 141)	92 (90, 110)	131 (110, 150)	0.012
Ischemia time, minutes	15 (9, 17)	23, 15, 30)	12.5 (12, 14)	17 (13, 20)	0.041
EBL, mL	100 (100, 250)	375 (100, 600)	150 (120, 180)	300 (150, 475)	0.047
eGFR, mL/min/1.73 m^2^ (POM3)	-6 (-9, -2)	0 (0, -1)	-4 (-6, 8)	-9 (-14, -4)	0.776
%Δ TGF-β1 urine level, ng/dL (POM3)	+ 8 (+ 3, + 11)	+ 18 (+ 12, + 19)	+ 8 (+ 2, + 10)	+ 18 (9, 37)	0.012
%Δ MCP-1 urine level, ng/dL (POM3)	+ 4 (0, + 5)	+ 11 (+ 5, + 36)	+ 2 (0, + 7)	+ 8 (+ 4, + 12)	0.017

Data are presented as medians (interquartile ranges)

*%Δ* percent difference from baseline, *EBL* estimated blood loss, *eGFR* estimated glomerular filtration rate, *MCP-1* monocyte chemoattractant protein 1, *POM* postoperative month, *TGF-β1* transforming growth factor beta 1

## Discussion

The optimal method for achieving tumor bed hemostasis during LPN remains an area of ongoing debate. Conventional double-layer suture renorrhaphy provides reliable hemostasis and collecting system closure but is associated with prolonged ischemia, parenchymal compression, and potential nephron loss [8, 13]. Laser-based coagulation offers an alternative approach that may reduce operative complexity and preserve renal parenchyma. While thulium lasers have demonstrated excellent cutting and coagulative properties in other urological applications [10, 17]. Their role in tumor bed coagulation during PN has not been prospectively studied. Our trial was designed to address this gap.

In this randomized study, we found that thulium beam coagulation significantly reduced operative time, WIT, blood loss, and suture use compared with standard suture renorrhaphy, without compromising perioperative safety. Functional outcomes at 3 months were comparable between groups, but biomarker analysis revealed a relatively low postoperative rises in urinary TGF-β1 and MCP-1 in the LLPN group, suggesting a lower fibrogenic response.

These findings highlight two important implications. First, the reduction in ischemia and blood loss reflects the efficient hemostatic profile of the thulium laser, which provides coagulation with minimal tissue penetration and limited collateral damage [17]. Second, the biomarker trends provide mechanistic support for improved parenchymal preservation. Renorrhaphy sutures inevitably devascularize adjacent tissue, promoting fibrosis and nephron loss [18, 19]. In contrast, surface coagulation avoids deep parenchymal injury and appears to blunt fibrotic remodeling, which may translate into better long-term function.

Although WIT was shorter in the LLPN group, correlation analysis across the entire cohort showed no significant association between ischemia duration and changes in fibrosis biomarkers. This suggests that the intergroup differences in biomarker response were not attributable to ischemia time but rather to reduced suture-related parenchymal injury with the laser technique.

Our results are consistent with previous reports suggesting that suture-free or simplified renorrhaphy techniques improve parenchymal preservation [20, 21]. Moreover, concerns raised with earlier lasers, such as holmium and diode systems, regarding smoke, poor visibility, and uncertain margins [22, 23], were not observed with thulium in our series. Consistent with Wang et al. [10] and Dubrovin et al. [24], we observed minimal smoke generation and reliable intraoperative visualization, allowing precise hemostasis.

At 3 months, the observed between-group difference in eGFR change was 4.39 ± 7.6 mL/min/1.73 m^2^. A post hoc power analysis based on this finding demonstrated 78% power (α = 0.05, two-tailed) and 82% power to detect a 5 mL/min/1.73 m^2^ difference. Although the study had adequate sensitivity for detecting moderate functional changes, smaller differences may have gone undetected because of the limited sample size and short follow-up. The trial, however, was primarily designed to evaluate perioperative outcomes and fibrosis biomarkers as early indicators of parenchymal injury that may precede later functional divergence. Renal function following partial nephrectomy can fluctuate over time, and progressive fibrosis may influence outcomes beyond the study period. While renal function recovery was comparable between groups at 3 months, the more favorable biomarker profile observed in the thulium laser group suggests a potential for improved long-term parenchymal preservation, warranting confirmation with extended follow-up.

Despite randomization, a higher proportion of right-sided tumors occurred in the CLPN group by chance. This imbalance could have increased procedural complexity and potentially biased perioperative outcomes in favor of the LLPN group. However, significant differences in ischemia time and biomarker response persisted after excluding these cases, supporting the reliability of the results.

The strengths of this trial include its prospective randomized design and the incorporation of mechanistic biomarker analysis, which provides insight into the biological effects of surgical technique. Limitations include the relatively small sample size, two-center setting, and short follow-up, which may limit generalizability and the ability to detect long-term functional differences. In addition, the cost of thulium laser technology may hinder widespread adoption.

## Conclusion

Our findings support thulium beam coagulation as a safe and effective alternative to double-layer suture renorrhaphy in LPN. By reducing ischemia, blood loss, and suture-related parenchymal injury, this technique may enhance nephron preservation without compromising surgical safety. Larger multicenter trials with longer follow-up are needed to validate these results and determine whether the observed biomarker differences translate into durable functional benefit.

## Data Availability

The datasets generated and analyzed during the current study are available from the corresponding author on reasonable request. All data have been anonymized to ensure patient confidentiality in accordance with institutional and ethical guidelines.
